# Genetic spectrum and clinical early natural history of glucose-6-phosphate dehydrogenase deficiency in Mexican children detected through newborn screening

**DOI:** 10.1186/s13023-021-01693-9

**Published:** 2021-02-26

**Authors:** Marcela Vela-Amieva, Miguel Angel Alcántara-Ortigoza, Ariadna González-del Angel, Leticia Belmont-Martínez, Carlos López-Candiani, Isabel Ibarra-González

**Affiliations:** 1 Laboratorio de Errores Innatos del Metabolismo Y Tamiz, Instituto Nacional de Pediatría SS, CDMX, Mexico; 2 Laboratorio de Biología Molecular, Instituto Nacional de Pediatría SS, CDMX, Mexico; 3 Servicio de Neonatología, Instituto Nacional de Pediatría SS, CDMX, Mexico; 4 UGN, Instituto de Investigaciones Biomédicas, UNAM-LEIMyT, Instituto Nacional de Pediatría SS, CDMX, Mexico

**Keywords:** Glucose-6-phosphate dehydrogenase deficiency, G6PD deficiency, Neonatal jaundice, Genetic disorders, Newborn screening, Hemolytic anemia, *UGT1A1*, Gilbert disease

## Abstract

**Background:**

Glucose-6-phosphate dehydrogenase deficiency (G6PDd) newborn screening is still a matter of debate due to its highly heterogeneous birth prevalence and clinical expression, as well as, the lack of enough knowledge on its natural history. Herein, we describe the early natural clinical course and the underlying *GDPD* genotypes in infants with G6PDd detected by newborn screening and later studied in a single follow-up center. G6PDd newborns were categorized into three groups: group 1: hospitalized with or without neonatal jaundice (NNJ); group 2: non-hospitalized with NNJ; and group 3: asymptomatic. Frequencies of homozygous UGT1A1*28 (rs34983651) genotypes among G6PDd patients with or without NNJ were also explored.

**Results:**

A total of 81 newborns (80 males, one female) were included. Most individuals (46.9%) had NNJ without other symptoms, followed by asymptomatic (42.0%) and hospitalized (11.1%) patients, although the hospitalization of only 3 of these patients was related to G6PDd, including NNJ or acute hemolytic anemia (AHA). Nine different G6PDd genotypes were found; the G6PD A^−202A/376G^ genotype was the most frequent (60.5%), followed by the G6PD A^−376G/968C^ (22.2%) and the Union-Maewo (rs398123546, 7.4%) genotypes. These genotypes produce a wide range of clinical and biochemical phenotypes with significant overlapping residual enzymatic activity values among class I, II or III variants. Some G6PD A^−202A/376G^ individuals had enzymatic values that were close to the cutoff value (5.3 U/g Hb, 4.6 and 4.8 U/g Hb in the groups with and without NNJ, respectively), while others showed extremely low enzymatic values (1.1 U/g Hb and 1.4 U/g Hb in the groups with and without NNJ, respectively). Homozygosity for UGT1A1*28 among G6PDd patients with (11.9%, N = 5/42) or without (10.3%, N = 4/39) NNJ did not shown significant statistical difference (p = 0.611).

**Conclusion:**

Wide variability in residual enzymatic activity was noted in G6PDd individuals with the same G6PD genotype. This feature, along with a documented heterogeneous mutational spectrum, makes it difficult to categorize G6PD variants according to current WHO classification and precludes the prediction of complications such as AHA, which can occur even with > 10% of residual enzymatic activity and/or be associated with the common and mild G6PD A^−376G/968C^ and G6PD A^−202A/376G^ haplotypes.

## Background

Glucose-6-phosphate dehydrogenase (G6PD, EC 1.1.1.49) is a cytosolic enzyme that catalyzes the first step of the pentose phosphate pathway to provide reduced equivalents to biosynthesis processes and to neutralize cell oxidative stress [1]. G6PD deficiency (G6PDd) is considered the most common human enzymopathy, which is inherited as a polymorphic X-linked trait attributed to nearly 230 hypomorphic variants in the *G6PD* gene (Xq28, MIM*305900) [2–4]. G6PDd affects more than 500 million people, although it has a worldwide distribution with very large variations in its prevalence ranging from zero in the original Amerindian populations to 20% in regions of Africa and Asia [4]. In addition, G6PDd has a great variety in its clinical expression, with most patients being asymptomatic, while others develop serious events of acute hemolytic anemia (AHA) that can be life-threatening or chronic [4, 5]. Neonatal jaundice (NNJ) is one of the clinical manifestations of G6PDd, and sometimes, its severity can lead to kernicterus [3, 6], although other genetic factors (i.e. (TA)_n_ promoter polymorphisms of *UGT1A1*, MIM*191740) could be influence the risk to develop hyperbilirubinemia in G6PDd neonates [7]. The WHO G6PDd classification from 1967 [8] establishes five classes of G6PDd based on the levels of enzyme residual activity determined in hemizygous males and according to associated clinical manifestations: class I: < 10% with chronic nonspherocytic hemolytic anemia (CNSHA) and acute exacerbations; class II: < 10% without clinical manifestations in the steady state; class III: 10–60% asymptomatic in the steady state; class IV: 100% asymptomatic; and class V: > 100% without clinical manifestations. However, Luzzato 2016 proposed a revised classification based on adult screening as follows: class I: residual activity < 10%; class II + III: < 30%; and class IV > 85% (with the elimination of class V) [6].

Particularly, population screening of G6PDd has been carried out in malaria endemic areas to prevent drug interactions that should trigger acute hemolytic crises in deficient individuals [9–11], but G6PDd mass newborn screening is still a matter of debate, and its implementation has been limited to few countries, mainly from Asia and Latin America [11–15]. Although some G6PDd newborn screening experiences in high-income countries such as Sweden have been reported [16], most of them do not include G6PDd detection in their recommended uniform screening panel [17], based on the argument of its highly heterogeneous birth prevalence and clinical expression as well as the lack of enough knowledge on its natural history [18].

Despite several publications regarding the results obtained for some G6PDd newborn screening programs, most of them are focused on its prevalence, cutoff and enzyme value distributions, and mutational spectrum [13–15, 19], but few of them try to establish the phenotype-genotype correlation [20], then reports about detailed clinical follow-up or medical interventions on G6PDd newborns are still limited.

In Mexico, a country with nearly 2 million births annually (https://www.inegi.org.mx/temas/natalidad/), the detection of G6PDd was added to the mandatory neonatal screening panel established by the Ministry of Health since 2015; this panel also includes congenital hypothyroidism, phenylketonuria, congenital adrenal hyperplasia, galactosemia and cystic fibrosis [21]. The first results of the G6PDd Mexican screening program confirmed its regional disparity in prevalence, ranging from 0.2 to 20%, as well as the identified difficulties in classifying affected patients [22].

The aim of this work is to report the G6PD activity levels, the underlying deficient G6PD genotypes, the phenotype-genotype correlation, and the early clinical characteristics documented in a group of Mexican infants with G6PDd, whose were detected by newborn screening and further evaluated in a single medical follow-up center.

## Methods

### Population study

**Fig. 1:**
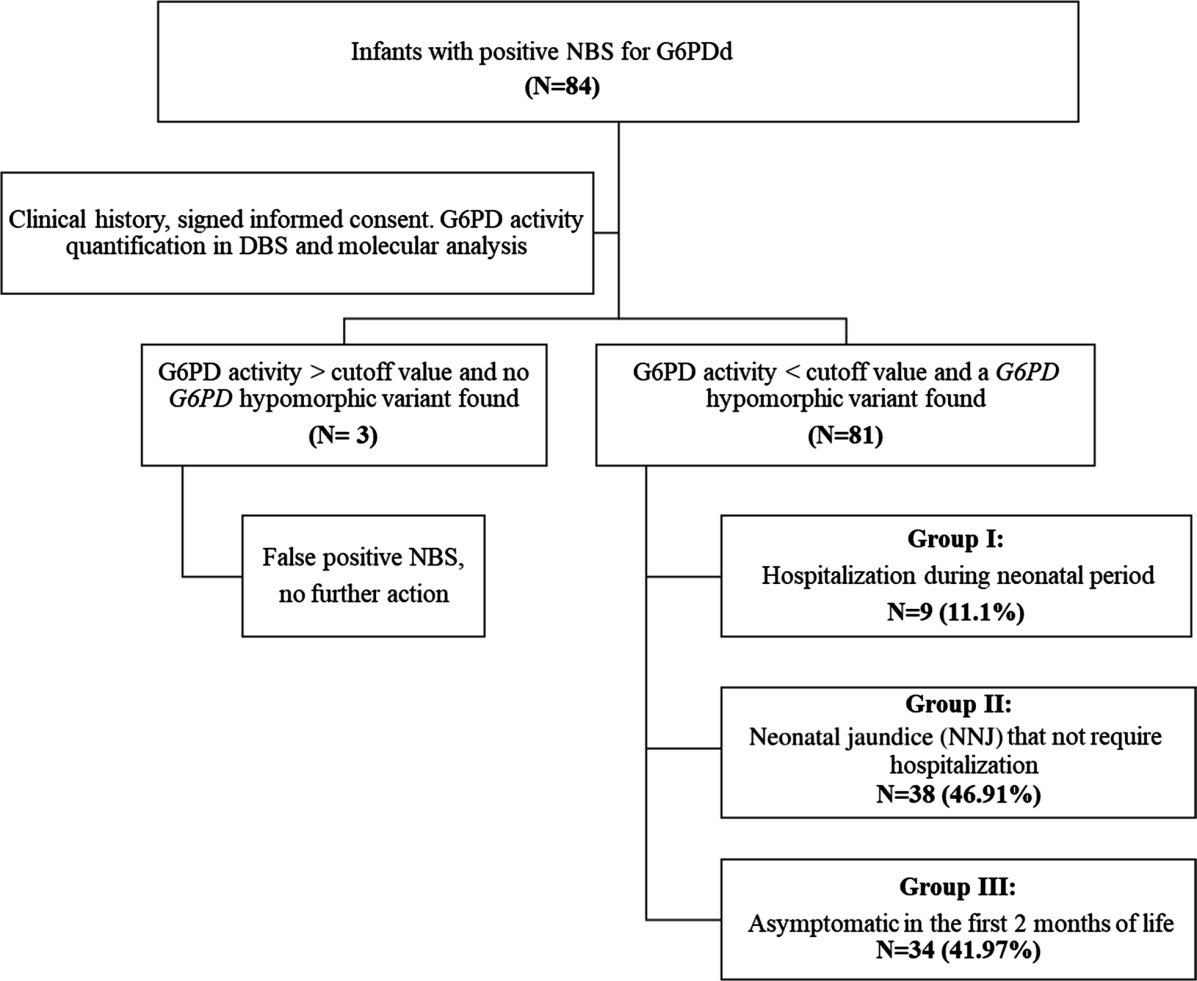
Study algorithm. Infants with a positive newborn screening (NBS) test came from different primary care health centers. The clinical and biochemical approach of the 84 unrelated individuals initially referred to our center as G6PDd started with a new measurement of the G6PD activity in dried blood spots (DBS) and further *G6PD* genotyping experiments that unequivocally confirmed the G6PDd status in 81 patients, with or without neonatal jaundice (NNJ)

Eighty-four infants (81 males, 3 females) with a suspicious (positive) result in the newborn screening for G6PDd were evaluated at the National Institute of Pediatrics from February 2018 to March 2020. Prematurity (< 37 weeks of gestation) was identified only in the 4.7% (N = 4/84) of included patients. The study algorithm is shown in Fig. 1. Confirmed patients were called for medical evaluation, including a record of risk factors (i.e., drugs or infections) and genetic counseling. False positive patients were informed and discharged to the first level attention medical units for healthy child control. Confirmed G6PDd patients were categorized as follows according to their clinical antecedents: group 1: hospitalized patients in the neonatal period with or without NNJ; group 2: non-hospitalized patients with NNJ; and group 3: asymptomatic newborns. Enzymatic and molecular studies were also offered for the siblings of G6PDd children. Clinical description was performed under the criteria of the Human Phenotype Ontology (HPO) database [23, 24; https://hpo.jax.org/app/]. The study was approved by the Institutional Research, Ethics and Biosecurity Boards (protocol registry 039/2018), and written informed consent was obtained from the parents of each participant.

### Blood samples for biochemical and genotype confirmation of G6PDd

Six drops of blood were extracted from each subject by heel puncture, deposited on a Guthrie card and allowed to dry for 3 h at room temperature. For older siblings, the sample was obtained by finger puncture.

### Confirmatory studies (short follow-up)

#### Enzyme activity quantification

To prevent G6PD activity decay in dried blood spots (DBS) [22], all the samples were stored at -20 °C and analyzed 48 h after extraction. G6PD activity was determined by the fluorometric method using commercial kits (test kit 6199860, LabSystems Diagnostics Oy, Vantaa, Finland). Briefly, 3 mm DBS disks were allocated into a 96-well microplate with calibrators and duplicate controls. A reaction mixture was reconstituted with a buffer solution. Then, 150 μL of the reaction mixture was added to each well. The plates were incubated for 30 min while being shaken. Then, 150 μL of cold copper reagent was added, and finally, the product of the enzymatic reaction was measured at an excitation wavelength of 355 nm and an emission wavelength of 460 nm. The cutoff value was established after enzymatic activity quantification of 564 DBS using the fifth percentile value of 5.3 U/g Hb.

#### G6PD molecular analysis

By processing 3–4 DBS punches of 3.2 mm in diameter, genomic DNA was obtained by the salting-out precipitation method (Gentra Puregene Blood Kit, QIAGEN, Hilden, Germany). PCR amplification of exons 3–4, 5, 6–7 and 9–10 of the *G6PD* gene (exon numbering and variant nomenclature according to NM_001042351.2), including their exon–intron boundaries, and further direct automated Sanger sequencing (primer and PCR conditions are available upon request) were applied to characterize the main hypomorphic haplotypes that account for ~ 90% of G6PDd alleles in the Mexican population [25]: G6PD A^−202A/376G^ (c.[202G>A;376A>G] or p.[Val68Met;Asn126Asp]), G6PD A^−376G/968C^ (c.[376A>G;968T>C] or p.[Asn126Asp;Leu323Pro]) and G6PD Santamaria^376G/542T^ (c.[376A>G;542A>T] or p.[Asn126Asp;Asp181Val]). The employed sequencing strategy allows the identification of other variants allocated at these *G6PD* gene regions and described previously as rare G6PDd variants in the Mexican population (i.e., Viangchan or p.(Val291Met) variant, rs137852327) [25]. In the patients with biochemically confirmed G6PDd but an initial normal Sanger sequencing result, further sequencing of exons 2, 8 and 11–13 of *G6PD* was subsequently applied to exclude the presence of other rare *G6PD* deficient variants, which have also been described in Mexican G6PDd patients (i.e., Union-Maewo or p.(Arg454Cys) variant, rs398123546). Whole *G6PD* sequencing was applied to confirm all suspected false-positive cases.

#### Genotyping of UGT1A1 (TA)n promoter variant

Homozygous frequency for the hypomorphic allele (A(TA)_7_TAA or UGT1A1*28 or NC_000002.12(NM_000463.2):c.-41_-40dup. rs34983651) associated to the reduced expression of *UGT1A1* gene and mainly responsible of Gilbert disease (MIM#143500), was assessed by means of PCR amplification and automated Sanger sequencing (primer and PCR conditions are available upon request) in all included G6PDd newborns. Homozygous A(TA)_7_TAA genotypic frequencies were compared by a chi square test among the G6PDd neonates with and without NNJ.

Continuous data are presented as medians with maximum and minimum values; categorical data are presented as counts and percentages. To establish differences between groups, one-way ANOVA was used. Data were analyzed using the R program (http://cran.r-project.org/).

## Results

A total of 81 patients (80 males and one female) showed enzymatic activity below the cutoff value (5.3 U/g Hb), so they were considered to have G6PDd and then subjected to molecular analysis. Three individuals (1 male and 2 females) had normal G6PD activity, and no hypomorphic variant was found after whole *G6PD* Sanger sequencing, so they were classified as false-positive patients. The number of patients in each clinical group is presented in Fig. 1, showing that the majority of individuals (46.9%, 38/81) had NNJ without other symptoms, 42.0% (34/81) were asymptomatic at the moment of the study, and 11.1% (9/81) were symptomatic patients (including NNJ) who required hospitalization. None of the hospitalized patients had been exposed to medications prior to their admission, and all were breastfed or receiving mixed feeding with human milk and starter infant formula. Moreover, the homozygous genotype for the A(TA)_7_TAA hypomorphic *UGT1A1* allele was found in 5/42 (11.9%) and 4/39 (10.3%) of G6PDd patients with or without NNJ, respectively. The chi square test did not reveal a significant statistical difference between the groups (*p* = 0.611).

Across the studied population, we found 9 different *G6PD* variants, all of which were previously described (Table 1). The most frequent deficient haplotype was G6PD A^−202A/376G^, which was found in 60.5% of the deficient patients, followed by G6PD A^−376G/968C^ which was found in 22.2% of the deficient patients, and Union-Maewo (p.(Arg454Cys) [rs398123546]), which was found in 7.4% of the deficient patients. The mean enzymatic activity of each variant and its geographical origin are shown in Table 1.

**Table 1:** Frequency of *G6PD* variants identified in the 81 Mexican G6PDd patients, according to WHO class and its mean enzymatic activity value

*G6PD* variant Legacy name	WHO class	*G6PD* genotype (according to NM_001042351.2)	Protein Change (according to NP_001035810.1)	Geographical origin	Present study		
					Frequency (%)	G6PD activity U/g Hb (min–max)	G6PD residual activity %
**Males (80)**							
G6PD A^−202A/376G^	III	c.[202G>A; 376A>G]	p.[Val68Met; Asn126Asp]	African	49 (60.5)	2.76 (1.1–4.8)	31.04
G6PD A^−376G/968C^	III	c.[376A>G; 968T>C]	p.[Asn126Asp; Leu323Pro]	African	18 (22.2)	1.55 (0.5–2.6)	17.37 (5.62–29.21)
Union-Maewo	II	c.[1360C>T]	p.[Arg454Cys]	Asian (Philippines)	6 (7.4)	0.07 (0.05–0.1)	0.73 (0.56–1.12)
Akrokorinthos	II–III	c.[463C>G]	p.[His155Asp]	Greece	2 (2.5)	3.33 (2.6–4)	37.36 (29.78–44.94)
Belem	II	c.[409C>T]	p.[Leu137Phe]	Brazil	1 (1.2)	1.1	12.36
Mediterranean	II	c.[563C>T]	p.[Ser188Phe]	Mediterranean	1 (1.2)	0.1	1.12
Santamaria^376G/542T^	II	c.[376A>G; 542A>T]	p.[Asn126Asp; Asp181Val]	Costa Rica, Italy	1 (1.2)	0.9	10.11
Mahidol	III	c.[487G>A]	p.[Gly163Ser]	Asian	1 (1.2)	2.7	30.34
Viangchan-Jammu	III	c.[871G>A]	p.[Val291Met]	Asian (China)	1 (1.2)	3.9	43.82
**Female (1)**							
Heterozygous G6PD A^−376G/968C^	III	c.[376A>G; 968T>C];[=]	p.[Asn126Asp; Leu323Pro];[=]	African	1 (1.2)	0.35	3.93

Of the 81 individuals, 9/81 (11.1%) patients, in accordance with the Luzzato 2016 classification [6], were class II, while 2/81 (2.4%) and 70/81 (86.4%) were class II-III and class III, respectively. However, there is an overlapping residual G6PD activity between classes II and III (Table 1).

The G6PD activity of the different clinical groups are shown in Table 2, while a detailed description of the patients who required hospitalization in the newborn period (Group 3) and its categorization according to the WHO and Luzzatto classification systems [6, 8] are shown in Table 3. The mean value of reticulocytes in the patients with NNJ was 2.22% (0.6–3.9) and in those without NNJ was 2.06% (0–4.8), and no significant difference was found between both groups (*p* = 0.376).

**Table 2:** G6PD activity and genotypes according to the different clinical groups

*G6PD* variant Legacy name	Relative proportion of patients^a^	Mean G6PD-Activity U/g Hb	Mean G6PD residual activity %^b^
**Group 1. Hospitalization during neonatal period n = 9**			
G6PD A^−202A/376G^	4/49	3 (1.55–4.60)	34.1 (17.42–51.66)
G6PD A^−376G/968C^	3/18	1.6 (1.2–2.0)	17.6 (13.48–22.47)
G6PD A^−202A/376G^ (Heterozygous female)	1/1	0.35	3.93
Mediterranean	1/1	0.10	1.12
Average (min-max)		**1.92 (0.10**–**4.60)**	**21.60 (1.12**–**51.69)**
**Group 2. NNJ non-hospitalized, n = 38**			
G6PD A^−202A/376G^	21/49	2.9 (1.10–4.10)	32 (12.36–46.07)
G6PD A^−376G/968C^	10/18	1.4 (0.50–2.33)	15.9 (5.62–26.22)
Union-Maewo	3/6	0.1 (0.05–0.10)	0.8 (0.78–1.12)
Akrokorinthos	1/1	2.7	29.8
Santamaria	1/1	0.9	10.1
Mahidol	1/1	2.7	30.3
Viangchan-Jammu	1/1	3.9	43.8
Average (min-max)		**2.22 (0.10–4.10)**	**24.97 (1.12–46.07)**
**Group 3. Asymptomatic during the neonatal period, n = 34**			
G6PD A^−202A/376G^	24/49	2.6 (1.40–4.80)	29.6 (15.73–53.93)
G6PD A^−376G/968C^	5/18	1.8 (1.20–2.60)	20.1 (13.48–29.21)
Union-Maewo	3/6	0.1 (0.05–0.06)	0.6 (0.56–0.67)
Akrokorinthos	1/1	4.0	44.9
Belem	1/1	1.1	12.4
Average (min-max)		**2.28 (0.05–4.8)**	**25.62 (0.56–53.93)**

Bold values indicate average (minimum-maximum)

^a^Denominator means the total number of patients of each variant

^b^There were no significant statistical differences among the three study groups (*P* = 0.707)

**Table 3:** Clinical description and type of *G6PD* variants found in the patients belonging to Group I (hospitalization during neonatal period)

ID	*G6PD* variant Legacy name	WHO class	Luzzato 2016 classification	G6PD activity U/g Hb	G6PD activity %	NNJ	Hospitalization				
							Clinical data (HPO ID)^a^	Age (days)	Cause	Cause related to G6PDd	Length (days)
30	Mediterranean	II	I	0.10	1.12	Yes	Seizures (HP: 0001250)	7	Treatment of seizures	No	7
71	G6PD A^−376G/968C^ (heterozygous female)	III	I	0.35	3.93	No	Neonatal asphyxia (HP: 0012768) Neonatal hypoglycemia (HP: 0001998)	< 1	Clinical surveillance	Unclear	3
14	G6PD A^−376G/968C^	III	II + III	1.20	13.48	Yes	NNJ (HP: 0000952)	< 1	Phototherapy	Yes	15
11^b^	G6PD A^−376G/968C^	III	II + III	1.50	16.85	Yes	NNJ (HP: 0000952)	7	Phototherapy	Yes	3
							AHA (HP: 0001878)	37	Anemia diagnostic approach requiring blood transfusion	Yes	4
2	G6PD A^−202A/376G^	III	II + III	1.55	17.42	No	Cough (HP: 0012735) Rhinitis (HP: 0012384) Fever (HP: 0001945) Neutropenia (HP: 0001875) Lethagy (HP: 0001254) AHA (HP: 0001878) Melanocitic nevus (HP: 0000995)	60	Upper respiratory tract infection, anemia diagnostic approach and transfusion	Yes	1
39	G6PD A^−376G/968C^	III	II + III	2.00	22.47	Yes	NNJ (HP: 0000952)	4	Phototherapy	Yes	1
15	G6PD A^−202A/376G^	III	Does not meet criteria^c^	3.00	33.71	No	Fever (HP: 0001945)	42	Diagnostic approach of sepsis	Unclear	3
57	G6PD A^−202A/376G^	III	II–III	3.00	33.71	Yes	NNJ (HP: 0000952) Tachypnea (HP: 0002789) Neonatal sepsis (HP: 0040187)	< 1	Sepsis diagnostic approach and NNJ treatment	Yes	15
19	G6PD A^−202A/376G^	III	Does not meet criteria	4.60	51.69	Yes	Transient apnea (HP: 0002104) Neonatal sepsis (HP: 00401879)	< 1	Diagnostic approach and sepsis treatment	Unclear	7

^a^HPO ID = The Human Phenotype Ontology (HPO, https://hpo.jax.org/app/) uses the latest Orphanet data and a different algorithm for ranking the differential diagnoses [23]

^b^Patient with 2 hospitalizations

^c^Does not meet criteria means that the observed residual activity is higher than that established by Luzzatto et al. [6]

**Fig. 2:**
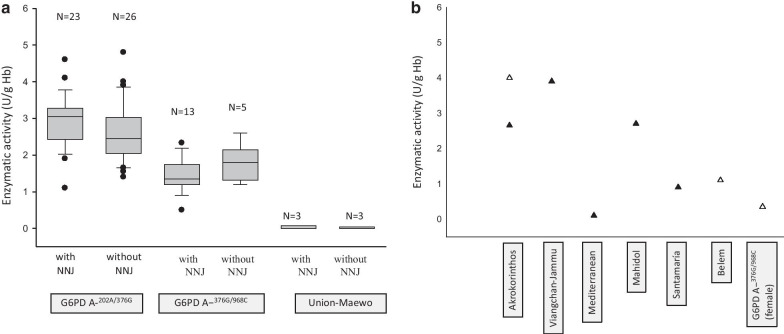
Documented G6PD deficient activity according to the identified *G6PD* patient genotypes (n = 81), with or without neonatal jaundice (NNJ). **a** Box and whisker plot genotypes with more than 3 affected individuals. No significant statistical differences were observed (*p* > 0.05); **b** Enzymatic activity documented in less than 3 affected individuals with corroborated *G6PD* genotypes. Filled triangles represent patients with NNJ, and open triangles are patients without NNJ

In four families, one or more siblings were G6PDd, and their results are presented in Table 4. In the studied newborns, 42/81 (51.85%) had NNJ, and 2/81 (2.47%) had hemolytic anemia. The residual enzymatic values according with the genotype and the presence of NNJ are shown in Fig. 2. The *G6PD* genotypes, enzymatic activity, and geographical origin of each of 81 patients are shown in Additional file 1: Table 1, and the proportion of patients with or without symptoms related to G6PDd, according to the genotype is presented in Additional file 1: Table 2.

**Table 4:** Residual G6PD activity and genotypes documented in the hemizygous siblings of the G6PDd newborn index patients

Patient	Relationship (age)	*G6PD* genotype (variant legacy name)	WHO class	% Residual enzymatic activity	Outcome until the time of this study
Family 1	Index newborn	Hemizygous G6PD A^−202A/376G^	III	1.4	Asymptomatic
	Half-brother (19 years old)	Hemizygous G6PD A^−202A/376G^	III	1.85	NNJ, healthy at the time of this study
Family 2	Index newborn	Hemizygous Mediterranean	II	0.1	NNJ, seizures
	Brother (15 years old)	Hemizygous Mediterranean	II	0.2	Healthy at the time of this study
Family 3	Index newborn	Hemizygous G6PD A^−202A/376G^	III	4	Asymptomatic
	Brother (3 years old)	Hemizygous G6PD A^−202A/376G^	III	2.8	NNJ requiring hospital management, AHA at 9 months and 2 years of age requiring blood transfusions
Family 4	Index newborn	Hemizygous G6PD A^−376G/968C^	III	1.35	Asymptomatic
	Brother (4 years old)	Hemizygous G6PD A^−376G/968C^	III	1.3	Asymptomatic

## Discussion

G6PDd is widely heterogeneous in terms of biochemical, clinical, and molecular manifestations [26]. In the present study, different G6PD mutations produced a wide range of clinical and biochemical phenotypes with significant overlapping residual enzymatic activity values between class I, II or III variants. Moreover, in the patients with the same genotype the residual enzymatic activity has no significant differences even in the presence or absence of NNJ (Fig. 2).

WHO classification has been established according to enzymatic activity and clinical severity of the patients, by assuming that class I patients are more prone to develop serious G6PDd clinical complications than those patients bearing class III *G6PD* variants. In class II newborns, the residual activity ranged from 0.56% to 12.36%, while in class III newborns, the residual activity ranged from 5.6 to 43.82%, and in a significant number of individuals, there was an overlap of the values (Table 1). However, our results revealed some discrepancies with the WHO classification, since the most severe patients with AHA were associated with class III genotypes (patients 2 and 11 bearing the G6PD A^−376G/968C^ and G6PD A^−202A/376G^ genotypes, respectively; Table 3). Usually, AHA is expected to be more common and severe in association with the Mediterranean variant than in the A^−^ variant [5], and then our results do not agree with this observation. Moreover, we did not find a correlation between the clinical severity and the WHO *G6PD* variant categorization. Significantly, only 7/9 patients could be categorized with the Luzzato classification, and the other two patients could not be classified as they did not meet the clinical or biochemical criteria (Table 3). Some authors have shown that G6PD enzymatic structural and functional activity of class II variants are more severe in vitro, suggesting a reclassification to a class I, but remarkably the blood samples came from apparently healthy donors without any symptoms related with G6PDd [27, 28]. This feature makes more complex the prediction of the clinical outcome of G6PDd patients and its genotype–phenotype correlation.

Several studies have revealed that the prevalence of G6PDd is higher in jaundiced newborns than in the control population, ranging from 8.9 to 28.1% [29–33]. Badejoko et al. found in a prospective observational study that 68.2% of G6PDd newborns presented with hyperbilirubinemia [34]. To our knowledge, the proportion of patients with NNJ in the reviewed publications on the results of G6PDd newborn screening is not stated. Instead, we found a high proportion of patients with NNJ (44/81, 54.32%), but only four of them (9%, 4/44) required hospitalization for jaundice management (Table 3). Even some individuals carrying the hypomorphic G6PD A^−202A/376G^ haplotype had enzymatic values that were close to the cutoff value (5.3 U/g Hb, 4.6 and 4.8 U/g Hb in the groups with and without NNJ, respectively), while other individuals showed extremely low enzymatic values (1.1 U/g Hb and 1.4 U/g Hb in the NNJ and without NNJ groups, respectively). In fact, we identified four G6PDd families with more than one affected patient (Table 4), in which only two patients had antecedent NNJ, and one of them had experienced two episodes of AHA at the ages of 9 and 24 months old, despite to carry a hemizygous G6PD A^−202A/376G^ genotype (class III) (Table 4). All these observations supported the idea that identical *G6PD* genotypes could lead to a wide range of phenotypes [4]; therefore, information that relies only on the *G6PD* genotype seems to be not useful for the prediction of clinical severity, as other causes, such as enzymatic kinetics or residual catalytic function related to structural stability [4, 6, 35]. Recently, Mansour-Hendili et al. [36] described patients with unexplained congenital hemolytic anemia that carried genetic variations in more than one gene, where G6PD variants were detected in combination with heterozygous β-spectrin, α-spectrin, solute carrier family 4 (anion exchanger) member 1, piezo-type mechanosensitive ion channel component 1, or hemoglobin-β locus, so other genetic as well as other epigenetic factors that are currently unknown, could be involved and must be further assessed. By assuming the low number of premature newborns (N = 4/84, 4.7%), all late preterm above 36 week’s gestation, a normal liver function would be expected, excluding prematurity as the main etiology of NNJ in the present study. Besides, the absence of significant statistical differences between the frequency of homozygous A(TA)_7_TAA *UGT1A1* genotypes among patients with or without NNJ, supports that Gilbert disease does not represent an important contribution to etiology of NNJ in our studied population, although strictly our analysis do not discard the possibility of other uncommon *UGT1A1* genotypes that can lead to unconjugated hyperbilirubinemia [i.e. compound heterozygous for Crigler-Najjar syndrome (MIM#218800, #606785) and A(TA)_7_TAA allele, or for the “G71R” (rs4148323) variant highly prevalent in Asian-derived populations [7]. Moreover, there were no significant differences among the enzymatic residual activity documented in the three groups of studies, although slightly lower enzymatic residual values were noted for newborns that required hospitalization (Table 2), neither Pearson test showed no correlation between the enzymatic G6PD activity and the reticulocytes count (*p* = 0.051). Nevertheless, one limitation of this study design is that it does not allow us to know the severe cases of kernicterus that could have occurred, which is to be expected in such patients that would be hospitalized or even deceased and not come to our newborn screening follow-up center.

Regarding the identified genotypic *G6PD* spectrum, the most frequently identified hypomorphic variants come from Africa (82.7% comprised by G6PD A^−202A/376G^ and G6PD A^−376G/968C^), followed by the class II Union-Maewo or p.(Arg454Cys) allele (7.4%) (Table 1), whose origin was presumably traced to the Philippines [13, 37]. Hemizygous Union-Maewo genotypes were found in six of our patients, and all of them showed the lowest enzymatic activity (mean 0.7, interval 0.05–0.1 U/g Hb), but only three of them had NNJ, and none of them required hospitalization, nor showed AHA. The Union-Maewo variant comprised 66% of G6PDd-responsible genotypes in patients who came from the Mexican Pacific coast (Guerrero, Supplemental Table 1), which could be a feature historically related to the intense commercial exchange (which included slave trade) established between the Philippines (Manila Galleon) and the Mexican Pacific coast during the sixteenth–seventeenth centuries [38]. The full sequencing of the coding region of the *G6PD* gene allowed for the identification of very rare variants, including the Mahidol, Belem and Akrokorinthos variants, which have been described mainly in specific populations from Thailand, Brazil and Greece, respectively [39–41]. To the best of our knowledge, we describe for the first time their presence in the Mexican population. Remarkably, none of the two patients with the Akrokorinthos variant had any known Greek or Mediterranean ancestry, and both families were originally from the state of Guerrero in the Pacific Coast. The patients with Belem and Mahidol variants came from the metropolitan area of Mexico City and denied having a known ancestry from Brazil or Southeast Asia, respectively (Supplemental Table 1). The identified heterogeneous genotypic spectrum may reflect the well-known multiethnicity of the Mexican population [42].

In summary, the present work shows that the same *G6PD* variant can lead to highly variable enzymatic residual activity as well as a wide phenotypic spectrum in the first month of life, which is in accordance with previously reported results [16]. The absence of an absolute or predictive phenotype-genotype correlation and the fact that almost 56% of the patients have symptoms related to G6PDd (Additional file 1: Tables 1 and 2), precludes the elaboration of guidelines on management, which agrees with statements of the ACMG NBS Expert Panel, who rejected the inclusion of G6PDd in the US newborn mandatory screening panel, due to the very limited data about the natural history of the disease, then encouraged the collection and publication of all the relevant clinical findings of the G6PDd screening programs [18]. Our work provides information on the early natural history of G6PDd newborns, and the present cohort will remain under surveillance.

## Conclusion

There is wide variability in the enzymatic activity in G6PDd individuals, even in those with the same *G6PD* genotype. This feature, along with a documented heterogeneous mutational spectrum, hinders the categorization of *G6PD* variants according to the current WHO classification and, importantly, precludes the prediction of complications such as AHA, which can occur even with > 10% of residual G6PD activity and/or associated with the common and mild G6PD A^−376G/968C^ and G6PD A^−202A/376G^ variants.

## Supplementary Information

**Additional file 1: Table 1.** Individual genotypes, geographic origin, residual enzymatic activity and concordance classification; **Table 2.** Proportion of patients with or without symptoms related to G6PDd, according to their genotype.

## Data Availability

The datasets analyzed during the present study are available from the corresponding author on reasonable request.

## Abbreviations

ACMG NBS: American College of Medical Genetics newborn screening panel
ASx: Asymptomatic
AHA: Acute hemolytic anemia
CNSHA: Chronic non spherocytic hemolytic anemia
DBil: Direct bilirubin
DBS: Dried blood sample
G6PD: Glucose-6-phosphate dehydrogenase
G6PDd: Glucose-6-phosphate dehydrogenase deficiency
Hb: Hemoglobin
HTC: Hematocrit
IBil: Indirect bilirubin
NADPH: Nicotinamide adenine dinucleotide phosphate
NNJ: Neonatal jaundice
Ret: Reticulocytes
TBil: Total bilirubin
WHO: World Health Organization
