# Supplementary material for: Genetic spectrum and clinical early natural history of glucose-6-phosphate dehydrogenase deficiency in Mexican children detected through newborn screening
Source: Orphanet J Rare Dis. 2021 Feb 26;16:103. doi: 10.1186/s13023-021-01693-9 (PMC7913327; doi:10.1186/s13023-021-01693-9)
Supplement: Supplementary file 1 — Additional file 1: Table 1. Individual genotypes, geographic origin, residual enzymatic activity and concordance classification; Table 2. Proportion of patients with or without symptoms related to G6PDd, according to their genotype. [file 13023_2021_1693_MOESM1_ESM.docx]

Supplementary Table 1. Individual genotypes, geographic origin, residual enzymatic activity and concordance classification

| ID patients | *G6PD* variant | Geografic origin | Enzyme activity in red cells (% of normal) | Clinical features | | Study group* | Classification according WHO 1967 | Genotype Phenotype Concordance |
| --- | --- | --- | --- | --- | --- | --- | --- | --- |
|  |  |  |  | AHA | NNJ |  |  |  |
| 30 | Mediterranean | CDMX | 1.1 | no | no | 1 | I | yes |
| 71 | G6PD A + (female) | Guanajuato | 3.9 | no | no | 1 | I | yes |
| 14 | G6PD A−^376G/968C^ | CDMX | 13.5 | no | yes | 1 | III | no |
| 11 | G6PD A−^376G/968C^ | CDMX | 16.9 | yes | yes | 1 | III | no |
| 2 | G6PD A^−202A/376G^ | CDMX | 17.4 | yes | no | 1 | III | no |
| 39 | G6PD A−^376G/968C^ | CDMX | 22.5 | no | yes | 1 | III | no |
| 15 | G6PD A^−202A/376G^ | Guerrero | 33.7 | no | no | 1 | III | no |
| 57 | G6PD A^−202A/376G^ | Edo. Mex | 33.7 | no | yes | 1 | III | no |
| 19 | G6PD A^−202A/376G^ | Michoacan | 51.7 | no | no | 1 | III | no |
| 34 | Unión-Maewo | Edo. Mex | 0.6 | no | yes | 2 | I | no |
| 17 | Unión-Maewo | Guerrero | 0.8 | no | yes | 2 | I | no |
| 10 | Unión-Maewo | Guerrero | 1.1 | no | yes | 2 | I | no |
| 74 | G6PD A−^376G/968C^ | Oaxaca | 5.6 | no | yes | 2 | I | no |
| 21 | Santamaría | Oaxaca | 10.1 | no | yes | 2 | III | no |
| 80 | G6PD A−^376G/968C^ | Guerrero | 11.2 | no | yes | 2 | III | no |
| 81 | G6PD A^−202A/376G^ | China | 12.4 | no | yes | 2 | III | no |
| 43 | G6PD A−^376G/968C^ | CDMX | 13.5 | no | yes | 2 | III | no |
| 55 | G6PD A−^376G/968C^ | Edo. Mex | 13.5 | no | yes | 2 | III | no |
| 58 | G6PD A−^376G/968C^ | Guerrero | 14.6 | no | yes | 2 | III | no |
| 65 | G6PD A−^376G/968C^ | CDMX | 15.2 | no | yes | 2 | III | no |
| 53 | G6PD A−^376G/968C^ | Guerrero | 16.9 | no | yes | 2 | III | no |
| 59 | G6PD A−^376G/968C^ | Guerrero | 18.5 | no | yes | 2 | III | no |
| 68 | G6PD A^−202A/376G^ | Guerrero | 21.3 | no | yes | 2 | III | no |
| 63 | G6PD A^−202A/376G^ | CDMX | 23 | no | yes | 2 | III | no |
| 31 | G6PD A^−202A/376G^ | Oaxaca | 23.6 | no | yes | 2 | III | no |
| 9 | G6PD A−^376G/968C^ | Guerrero | 24.2 | no | yes | 2 | III | no |
| 16 | G6PD A^−202A/376G^ | CDMX | 24.7 | no | yes | 2 | III | no |
| 6 | G6PD A−^376G/968C^ | Oaxaca | 26.2 | no | yes | 2 | III | no |
| 7 | G6PD A^−202A/376G^ | Edo. Mex | 27 | no | yes | 2 | III | no |
| 51 | G6PD A^−202A/376G^ | CDMX | 28.1 | no | yes | 2 | III | no |
| 18 | G6PD A^−202A/376G^ | Guerrero | 29.2 | no | yes | 2 | III | no |
| 47 | Acrokorinthos | Guerrero | 29.8 | no | yes | 2 | III | no |
| 36 | Mahidol | CDMX | 30.3 | no | yes | 2 | III | no |
| 35 | G6PD A^−202A/376G^ | CDMX | 33.7 | no | yes | 2 | III | no |
| 48 | G6PD A^−202A/376G^ | Hidalgo | 33.7 | no | yes | 2 | III | no |
| 22 | G6PD A^−202A/376G^ | Guerrero | 34.3 | no | yes | 2 | III | no |
| 27 | G6PD A^−202A/376G^ | Veracruz | 34.8 | no | yes | 2 | III | no |
| 32 | G6PD A^−202A/376G^ | CDMX | 34.8 | no | yes | 2 | III | no |
| 54 | G6PD A^−202A/376G^ | CDMX | 36 | no | yes | 2 | III | no |
| 76 | G6PD A^−202A/376G^ | CDMX | 36 | no | yes | 2 | III | no |
| 77 | G6PD A^−202A/376G^ | Edo. Mex | 36 | no | yes | 2 | III | no |
| 49 | G6PD A^−202A/376G^ | Guerrero | 37.1 | no | yes | 2 | III | no |
| 70 | G6PD A^−202A/376G^ | CDMX | 39.3 | no | yes | 2 | III | no |
| 28 | G6PD A^−202A/376G^ | Guerrero | 40.4 | no | yes | 2 | III | no |
| 12 | G6PD A^−202A/376G^ | Edo. Mex | 41.6 | no | yes | 2 | III | no |
| 79 | Viangchan-Jammu | Edo. Mex | 43.8 | no | yes | 2 | III | no |
| 75 | G6PD A^−202A/376G^ | Guerrero | 46.1 | no | yes | 2 | III | no |
| 50 | Unión-Maewo | Guerrero | 0.6 | none | | 3 | I | no |
| 8 | Unión-Maewo | Guerrero | 0.7 | none | | 3 | I | no |
| 56 | Unión-Maewo | Edo. Mex | 0.7 | none | | 3 | I | no |
| 41 | Belem | CDMX | 12.4 | none | | 3 | III | yes |
| 13 | G6PD A−^376G/968C^ | Edo. Mex | 13.5 | none | | 3 | III | yes |
| 72 | G6PD A−^376G/968C^ | CDMX | 15.2 | none | | 3 | III | yes |
| 26 | G6PD A^−202A/376G^ | Edo. Mex | 15.7 | none | | 3 | III | yes |
| 3 | G6PD A^−202A/376G^ | Edo. Mex | 18.5 | none | | 3 | III | yes |
| 45 | G6PD A^−202A/376G^ | CDMX | 19.1 | none | | 3 | III | yes |
| 33 | G6PD A^−202A/376G^ | CDMX | 20.2 | none | | 3 | III | yes |
| 73 | G6PD A−^376G/968C^ | Guerrero | 20.2 | none | | 3 | III | yes |
| 61 | G6PD A^−202A/376G^ | CDMX | 22.1 | none | | 3 | III | yes |
| 46 | G6PD A−^376G/968C^ | CDMX | 22.5 | none | | 3 | III | yes |
| 67 | G6PD A^−202A/376G^ | Edo. Mex | 23 | none | | 3 | III | yes |
| 37 | G6PD A^−202A/376G^ | CDMX | 24.2 | none | | 3 | III | yes |
| 60 | G6PD A^−202A/376G^ | Edo. Mex | 24.7 | none | | 3 | III | yes |
| 25 | G6PD A^−202A/376G^ | CDMX | 25.3 | none | | 3 | III | yes |
| 38 | G6PD A^−202A/376G^ | Edo. Mex | 25.8 | none | | 3 | III | yes |
| 62 | G6PD A^−202A/376G^ | CDMX | 25.8 | none | | 3 | III | yes |
| 4 | G6PD A^−202A/376G^ | Edo. Mex | 27 | none | | 3 | III | yes |
| 5 | G6PD A^−202A/376G^ | Guerrero | 28.1 | none | | 3 | III | yes |
| 69 | G6PD A^−202A/376G^ | Edo. Mex | 28.7 | none | | 3 | III | yes |
| 64 | G6PD A−^376G/968C^ | CDMX | 29.2 | none | | 3 | III | yes |
| 23 | G6PD A^−202A/376G^ | Edo. Mex | 30.3 | none | | 3 | III | yes |
| 24 | G6PD A^−202A/376G^ | CDMX | 31.5 | none | | 3 | III | yes |
| 40 | G6PD A^−202A/376G^ | Edo. Mex | 33.7 | none | | 3 | III | yes |
| 66 | G6PD A^−202A/376G^ | CDMX | 34.1 | none | | 3 | III | yes |
| 44 | G6PD A^−202A/376G^ | Edo. Mex | 36 | none | | 3 | III | yes |
| 78 | G6PD A^−202A/376G^ | Guerrero | 36 | none | | 3 | III | yes |
| 42 | G6PD A^−202A/376G^ | Guerrero | 38.8 | none | | 3 | III | yes |
| 1 | G6PD A^−202A/376G^ | Guerrero | 43.8 | none | | 3 | III | yes |
| 29 | Acrokorinthos | Guerrero | 44.9 | none | | 3 | III | yes |
| 52 | G6PD A^−202A/376G^ | Tlaxcala | 44.9 | none | | 3 | III | yes |
| 20 | G6PD A^−202A/376G^ | Guerrero | 53.9 | none | | 3 | III | yes |

*Group 1: Hospitalized G6PDd patients in the neonatal period, with or without NNJ; Group 2: Non-hospitalized G6PDd patients with NNJ; Group 3: asymptomatic G6PDd newborns.

Supplementary Table 2. Proportion of patients with or without symptoms related to G6PDd, according to their genotype

| Genotype | N | With G6PDd related symptoms, N (%) | Without G6PDd related symptoms, N (%) |
| --- | --- | --- | --- |
| G6PD A−^202A/376G^ | 49 | 24 (49) | 25 (51) |
| G6PD A−^376G/968C^ | 18 | 13 (72) | 5 (28) |
| Union-Maewo | 6 | 3 (50) | 3 (50) |
| Akrokorinthos | 2 | 1 (50) | 1 (50) |
| Santamaria | 1 | 1 (100) | 0 |
| Mahidol | 1 | 1 (100) | 0 |
| Viangchan-Jammu | 1 | 1 (100) | 0 |
| Mediterranean | 1 | 1 (100) | 0 |
| Belem | 1 | 0 | 1 (100) |
| G6PD A−^202A/376G^ (Heterozygous female) | 1 | 0 | 1 (100) |
| **TOTAL** | **81** | **45 (56)** | **36 (44)** |
